# Text recycling: acceptable or misconduct?

**DOI:** 10.1186/s12916-014-0148-8

**Published:** 2014-08-16

**Authors:** Stephanie Harriman, Jigisha Patel

**Affiliations:** BioMed Central, Floor 6, 236 Gray’s Inn Road, London, WC1X 8HB UK

**Keywords:** Text recycling, Self-plagiarism, Publication ethics, Transparency, Guidelines

## Abstract

Text recycling, also referred to as self-plagiarism, is the reproduction of an author’s own text from a previous publication in a new publication. Opinions on the acceptability of this practice vary, with some viewing it as acceptable and efficient, and others as misleading and unacceptable. In light of the lack of consensus, journal editors often have difficulty deciding how to act upon the discovery of text recycling. In response to these difficulties, we have created a set of guidelines for journal editors on how to deal with text recycling. In this editorial, we discuss some of the challenges of developing these guidelines, and how authors can avoid undisclosed text recycling.

The guidelines can be found here: http://media.biomedcentral.com/content/editorial/BMC-text-recycling-editorial_guidelines.pdf

## Background

Imagine yourself as a journal editor. You find that a manuscript submitted to your journal includes text reproduced verbatim from another publication by the same authors. How do you view this? Is it wrong of the authors to copy and paste their own words? Do you see it as misleading and deceitful, or an acceptable and efficient action?

Of course, in reality, the question is not this simple. There are many, sometimes conflicting, factors that may motivate an author to reproduce parts of their own previously published text, a practice known as text recycling. As an editor, these factors, not least what is being copied and whether the authors are transparent, may influence your judgement. So, how would you judge and how would you maintain consistency in your judgement?

The need for guidelines to deal with this issue has been raised by editors. Having dealt with many cases of text recycling ourselves, we too were aware that this practice is viewed by some as totally justified, harmless and acceptable, while at the same time felt unease that in some circumstances it didn’t seem ‘right’. There followed a series of discussions, including at the Committee on Publication Ethics (COPE) forum [1], opinion seeking, drafts and re-drafts which have culminated in our ‘text recycling guidelines’ for editors [2] and an editorial policy on text recycling to ensure authors’ awareness [3].

## The challenges

These new guidelines are not intended as the definitive last word on the subject, but rather a work in progress. We are aware that there are still some areas where opinions will differ.

One area of disagreement we encountered was the use of the term ‘text recycling’ rather than ‘self-plagiarism’. We have chosen the term ‘text recycling’ to separate the issue from plagiarism of other people’s work, which is always unacceptable and constitutes misconduct. Some disagree with this terminology and feel that ‘self-plagiarism’ better describes what they see as an unacceptable practice, and feel that referring to it as ‘recycling’ makes it sound desirable.

Another key issue in the development of the guidelines was that opinions differ on the extent to which text recycling is viewed as acceptable. The general consensus among those who took part in our discussions [1] was that it is acceptable under some circumstances and sometimes even desirable: for example, where authors have repeated a method from a previous study. Not only may there be very limited ways to describe it but, provided the original article is cited, it is more transparent and ‘reader friendly’ if it is described in exactly the same way so it is clear that the same method was used. Text recycling, with appropriate citation, may also be acceptable in the introduction to introduce background ideas that have been discussed in related articles. Conversely, overlap in the results section will often constitute duplicate publication and is nearly always unacceptable. We also argue that it is seldom acceptable in the conclusions as these should focus on the novel aspects of the article.

As is so often the case, the key is transparency – authors should be clear in all cases where there is overlap, by both citing re-used text in the manuscript and alerting the editor on submission.

Another point that was debated while formulating the guidelines was the need to distinguish text recycling from duplicate publication. Duplicate (also referred to as redundant) publication refers to substantial (or in some cases complete) overlap in data/results. Duplicate publication is a serious form of misconduct and an issue for which there are already clear guidelines from COPE [4].

The final point of contention was how far back to apply the guidelines. Over time, the ability to detect overlapping text has increased. Many journals now use plagiarism detection software, which often detects text recycling. Attitudes towards the reuse of one’s own words in a subsequent publication may also have become more stringent. There is no recommended cut-off date before which the guidelines do not apply, but for older published articles, editors should take into account standards and accepted norms at the time of publication.

## Conclusions

The development of online publishing, plagiarism detection software and publishing standards have increased awareness of text recycling and highlighted issues where editors may well have cause for concern. We do not view text recycling as wrong or unethical *per se* and acknowledge that there are circumstances where it is completely valid and appropriate to re-use one’s own text. While the problem of how to deal with text-recycling has primarily troubled editors, authors too may wonder how far it is acceptable to re-use their previously published text. Although different journals will have different policies, we hope these guidelines will be a useful source of guidance for authors who feel they have a justifiable reason to re-use their previously published text as well as for editors. We welcome feedback from authors and editors on how the guidelines can be improved.
